# Investigation on the Alteration of Brain Functional Network and Its Role in the Identification of Mild Cognitive Impairment

**DOI:** 10.3389/fnins.2020.558434

**Published:** 2020-09-30

**Authors:** Lulu Zhang, Huangjing Ni, Zhinan Yu, Jun Wang, Jiaolong Qin, Fengzhen Hou, Albert Yang, Alzheimer’s Disease Neuroimaging Initiative (ADNI)

**Affiliations:** ^1^Key Laboratory of Biomedical Functional Materials, School of Science, China Pharmaceutical University, Nanjing, China; ^2^Smart Health Big Data Analysis and Location Services Engineering Lab of Jiangsu Province, School of Geographic and Biologic Information, Nanjing University of Posts and Telecommunications, Nanjing, China; ^3^Key Laboratory of Intelligent Perception and Systems for High-Dimensional Information of Ministry of Education, School of Computer Science and Engineering, Nanjing University of Science and Technology, Nanjing, China; ^4^Division of Interdisciplinary Medicine and Biotechnology, Department of Medicine, Beth Israel Deaconess Medical Center/Harvard Medical School, Boston, MA, United States

**Keywords:** Alzheimer’s disease, mild cognitive impairment, resting-state functional MRI, modular structure, graph theory, machine learning

## Abstract

Mild cognitive impairment (MCI) is generally regarded as a prodromal stage of Alzheimer’s disease (AD). In coping with the challenges caused by AD, we analyzed resting-state functional magnetic resonance imaging data of 82 MCI subjects and 93 normal controls (NCs). The alteration of brain functional network in MCI was investigated on three scales, including global metrics, nodal characteristics, and modular properties. The results supported the existence of small worldness, hubs, and community structure in the brain functional networks of both groups. Compared with NCs, the network altered in MCI over all the three scales. In scale I, we found significantly decreased characteristic path length and increased global efficiency in MCI. Moreover, altered global network metrics were associated with cognitive level evaluated by neuropsychological assessments. In scale II, the nodal betweenness centrality of some global hubs, such as the right Crus II of cerebellar hemisphere (CERCRU2.R) and fusiform gyrus (FFG.R), changed significantly and associated with the severity and cognitive impairment in MCI. In scale III, although anatomically adjacent regions tended to be clustered into the same module regardless of group, discrepancies existed in the composition of modules in both groups, with a prominent separation of the cerebellum and a less localized organization of community structure in MCI compared with NC. Taking advantages of random forest approach, we achieved an accuracy of 91.4% to discriminate MCI patients from NCs by integrating cognitive assessments and network analysis. The importance of the used features fed into the classifier further validated the nodal characteristics of CERCRU2.R and FFG.R could be potential biomarkers in the identification of MCI. In conclusion, the present study demonstrated that the brain functional connectome data altered at the stage of MCI and could assist the automatic diagnosis of MCI patients.

## Introduction

Alzheimer’s disease (AD), a neurodegenerative disease, represents the most common type of dementia (Ahmadlou et al., 2010; Li et al., 2011). The prevalence of AD is a tremendous burden to individuals, families, and society. The treatment for AD remains unavailable and no way can prevent or reverse the progression of AD; only the early intervention of AD may influence its onset and deterioration (Al-Shoukry et al., 2020). Mild cognitive impairment (MCI) is generally regarded as a prodromal stage of AD since patients with MCI convert to AD at a rate of approximately 15% per year (Davatzikos et al., 2011). Hence, it is important to explore the neuropathological alteration in MCI, discover potential target for neuromodulation in treating MCI, and prompt effective method for the early diagnosis of MCI.

Theoretically, the human brain can be represented as a “connectome,” a large-scale network of interconnected regions that provides the anatomical substrate for neural communication, functional processing, and information integration (Fornito et al., 2013). Brain connectivity has been widely analyzed based on the graph theory by regarding neural elements (e.g., neurons and brain regions) as nodes and some measures of structural, functional, or causal interaction between nodes as edges (Fornito et al., 2013; Onias et al., 2014; Khazaee et al., 2015; Fang et al., 2017). Numerous studies have demonstrated its effectiveness in investigating the altered brain network pattern with MCI (Yao et al., 2010, 2018; Zhao et al., 2012; Seo et al., 2013; Xiang et al., 2013; Son et al., 2015; Deng et al., 2016; Pereira et al., 2016; Sánchez-Catasús et al., 2018). One of the shortcomings in most related studies is that only the cerebral regions were considered. However, recent studies have demonstrated that the cerebellum may play a vital role in neurodegenerative processes like AD. Evidences from functional imaging studies have reported the involvement of the cerebellum in various cognitive tasks besides the traditional motor ones (Stoodley, 2012), and cerebellar abnormality has also been reported in AD/MCI patients recently (Tabatabaei-Jafari et al., 2017; Pagen et al., 2020). Therefore, exploring the whole-brain functional network, including both cerebral and cerebellar regions, can disclose more comprehensive information of the abnormal brain connectome in MCI patients.

Graph metrics of the functional brain network are found to be informative to characterize MCI patients (Khazaee et al., 2016; Xu et al., 2020). In practice, in a brain functional network, a region can be deemed as a node, and the edges can be determined by the functional interaction of nodes. Various metrics have been proposed in the literature to quantify the topological characteristics of such a network and can be generally classified into three distinct scales. Measures from the three scales variously focus on characterizing aspects of function integration and segregation, quantifying importance of individual brain regions, and detecting patterns of local anatomical circuitry (Rubinov and Sporns, 2010; Tijms et al., 2013).

Moreover, the past decades witness the increasing growth in clinical use of artificial intelligence. Machine learning approaches are found to be quite useful for discriminating MCI patients from normal controls (NCs) (Tanveer et al., 2020). Some researchers made use of linguistic and/or acoustic features (Gosztolya et al., 2019; Orimaye et al., 2020; Calzà et al., 2021), while the overwhelming majority of previous studies focused on utilizing the neuroimaging biomarkers for the identification of MCI (Tanveer et al., 2020). Growing functional magnetic resonance imaging (fMRI) studies have been devoted to the classification task between MCI patients and NCs (Chen et al., 2011; Jie et al., 2013, 2014; Suk et al., 2013; Wang et al., 2013; Wee et al., 2013a,b; Cui et al., 2018; Xu et al., 2020). Most recently, Xu et al. (2020) utilized a combination of information in the functional brain connectome for the discrimination of MCI and NC. When the functional connections, global metrics, and nodal metrics were combined, an accuracy of 92.9% was achieved on 105 participants (41 MCI patients and 60 NCs). However, accuracy dramatically dropped to 66.0% when testing the pretrained model with an independent dataset from the AD Neuroimaging Initiative (ADNI) database (Xu et al., 2020).

In this study, we retrospectively analyzed resting-state fMRI (rs-fMRI) data derived from 82 MCI patients and 93 NCs from ADNI. Brain functional networks were constructed from rs-fMRI data, and the network metrics were analyzed from three scales. Both cerebral and cerebellar regions were covered in the construction of the graph. A weighted network was used in order to keep the information in the functional connectivity (FC). Furthermore, graph metrics were then combined to train and validate an automatic model on MCI and NC subjects. Our primary goal was to investigate the alterations of network properties that occurred at the stage of MCI and to find out whether the analysis of functional network can assist the accurate diagnosis of MCI patients.

## Participants

In this study, rs-fMRI data derived from 82 MCI patients and 93 NCs were obtained from the ADNI database.^1^ Participants in ADNI were included in the present study if they met the following criteria: (i) ages between 55.0 and 80.0; (ii) scanning with parameters of repetition time of 3,000 ms, echo time of 30 ms, flip angle of 80° or 90°, slices of 48, and voxel size of 3.31 mm × 3.31 mm × 3.31 or 3.44 mm × 3.44 mm × 3.40 mm; (iii) available records of their cognitive and behavioral assessments, comprising scores from the 13-item AD assessment scale (ADAS13), clinical dementia rating scale sum of boxes (CDRSB), Mini-Mental State Examination (MMSE), and frequently asked questions (FAQ); (iv) head motions <1.5 mm and 1.5°; (v) mean fractional displacement head motion values <0.2 mm; and (vi) satisfying MRI quality control and excluding unclear spatial normalization pictures. The demographics and clinical characteristics of the participants are illustrated in Table 1. Cross-sectional comparisons indicated a significant group effect on ADAS13, CDRSB, MMSE, or FAQ scores.

**TABLE 1:** Demographic and clinical characteristics of MCI and NC.

**Information**	**NC**	**MCI**	***p*-value**
Number of participants	93	82	
Age (years)	70.47 ± 5.91	71.61 ± 5.1	0.176
Gender (male/female)	46/36	36/57	0.024*
ADAS13 score	14.92 ± 6.81	11.62 ± 5.3	0.001*
CDRSB score	1.38 ± 1.26	0.13 ± 0.6	<0.001*
MMSE score	27.89 ± 1.82	28.88 ± 1.46	<0.001*
FAQ total score	3.15 ± 4.53	0.4 ± 1.93	<0.001*

NC, normal controls; MCI, mild cognitive impairment; ADAS13, 13-item Alzheimer’s Disease Assessment Scale; CDRSB, Clinical Dementia Rating sum of boxes score; MMSE, Mini-Mental State Examination; FAQ, frequently asked questions; plus–minus score values are mean ± SD. *Significant difference between the two groups (*p* < 0.05).

### Data Preprocessing

In this study, after discarding the first several volumes (five and seven for the data acquired before and after year 2014, respectively), data preprocessing was conducted with the help of Data Processing Assistant for Resting-State fMRI Advanced Edition (version 4.3), which is based on Statistical Parametric Mapping (SPM,^2^) and the toolbox for Data Processing and Analysis of Brain Imaging (DPABI^3^) (Yan and Zang, 2010; Yan et al., 2016). First, slice timing, motion correction, and normalization to the Montreal Neurological Institute space were conducted using T1 image unified segmentation. Then, nuisance covariates including six head motion parameters, white matter signal, cerebral spinal fluid signal, and global signal were regressed. In order to remove the spiking influence caused by motion artifacts (Power et al., 2012; Burgess et al., 2016; Ciric et al., 2017, 2018), a despiking step (Parkes et al., 2017) was adopted. Next, the temporal filtering (0.01–0.1 Hz) step was performed. Like general fMRI data using the echo-planar imaging sequence, artifacts could be caused by the distortion and loss of signal in the anterior temporal and orbitofrontal regions in ADNI, which might influence the connectivity between these regions and the others. To reduce the variability due to susceptibility artifacts, temporal signal-to-noise ratio (TSNR) (Murphy et al., 2007) map on the whole brain was calculated for each subject. A binary TSNR mask was obtained when a threshold of 20 was set on the averaged TSNR map (Zhuo et al., 2016) and further intersected with the Automated Anatomical Labeling (AAL) atlas to generate the final mask. Finally, based on the TSNR-thresholded AAL atlas, the preprocessed images were parcellated into 116 regions of interest (ROIs) and the regional mean time series of blood oxygenation level-dependent signals with the first 135 time points were extracted from each ROI for the further analysis.

### From ROI Time Series to Weighted and Undirected Network

Time series derived from the *i*th and *j*th ROIs were denoted as *X*_*i*_ and *X*_*j*_, respectively. The absolute Pearson correlation coefficient between *X*_*i*_ and *X*_*j*_, denoted as *r*_*ij*_ and calculated by Formula (1), was used in the present study to evaluate the FC between the two ROIs.

(1) $$r_{i\,j}=\left|\frac{\sum \left(X_{i}-\bar{X_{i}}\right)\,\left(X_{j}-\bar{X_{j}}\right)}{\sqrt{\sum {\left(X_{i}-\bar{X_{i}}\right)}^{2}}\,\sqrt{\sum {\left(X_{j}-\bar{X_{j}}\right)}^{2}}}\right|$$

Here, $\bar{X_{i}}$ and $\bar{X_{j}}$ represent the mean of time series *X*_*i*_ and *X*_*j*_, respectively. Given a total of *N* ROIs, a symmetric matrix ***w*** with *N*^∗^*N* elements can be obtained by evaluating the FC values over all the possible ROI pairs, as shown in Formula (2):

(2) $$w_{i\,j}=\left\{ \begin{array}{cc} 1, & i=j\\ r_{i\,j}, & i,j\in \left[1,N\right],\text{and}\,i\neq j \end{array}\right.$$

In order to exclude the self-connections, values in the diagonal line of matrix ***w*** were then set to 0. In this way, for each individual, a fully connected, undirected, and weighted network was obtained by regarding each ROI as a node and ***w*** as the adjacent matrix. We also constructed group-level networks based on the individual adjacent matrix for MCI patients and NCs, respectively. That is, the element ***w*_*ij*_** of the group-level network is the average value of ***w*_*ij*_** in each individual graph within a certain group. Such a group-level network summarizes FC maps on average over all subjects within the group and captures the connectivity backbone of the group (Meunier et al., 2009; Sun et al., 2014).

### Topological Metrics in Three Scales

The topological characteristics of a fully connected network might be contaminated by the presence of numerous weak connections among ROIs. Generally, a threshold is used, and only the supra-threshold FCs are retained, leading to a sparse network for analysis. The term network sparsity or density was proposed to represent the proportion of supra-threshold connections relative to all possible connections. As most graph theoretic measures are contingent on the number of nodes and the connection density, it is common to prescribe a shared network sparsity for all the networks compared (Fornito et al., 2013). However, there is no unified rule for the determination of network sparsity. Therefore, we used a wide range of sparsities, i.e., from 5 to 50% with steps of 1%, to analyze graphical properties of brain functional network. When a certain sparsity was used, each full-connected network (estimated for either an individual or a group) was thresholded by keeping the corresponding number of edges with the strongest FCs.

For each participant, based on the individual network thresholded by a certain sparsity, classical network metrics for scale I, such as the clustering coefficient (*C*), characteristic path length (*L*), global efficiency (*GE*), and small worldness (*SW*, random number was set as 1,000) were investigated in this study. In scale II, the regional nodal characteristics regarding the global hubs were assessed qualitatively on the group-level networks obtained across the sparsities ranging from 5 to 50%. The betweenness centrality of a node *i* (denoted as *bc*_*i*_) in the group-level network was calculated and normalized as *BC*_*i*_ = *bc*_*i*_/ < *bc*_*i*_ >, where < *bc*_*i*_ > is the average betweenness of all nodes. *BC*_*i*_ measures the importance of node *i* over information flow between other nodes throughout the network, and the regions with high values of *BC*_*i*_ are usually identified as hubs. Some studies identified hubs as nodes with *BC*_*i*_ larger than 1.5 (He et al., 2008) or 2 (Yao et al., 2010), and in this study, we used a stricter threshold for the definition of hubs as 2.5. To be noted, nodal *bc*_*i*_ was also calculated for each subject on the individual network and the group difference was evaluated.

In scale III, we investigated the modular structure quantitatively via the group-level networks. The modular organization has been thought to be one of the most fundamental principles in complex systems and demonstrated to exist in human brain networks in previous studies (Deng et al., 2016; Pereira et al., 2016; Jalili, 2017). Modularity (denoted as Q in the following), a measure for the quality of the community structure in a network (Newman, 2006), was computed on the group-level networks for a qualitative assessment when network sparsity ranged from 5 to 50%, with steps of 1%. Meanwhile, modules were detected as subsets of nodes in the network that are more densely connected to the other nodes in the same module than to nodes outside the module (Radicchi et al., 2004).

Definitions and brief descriptions of the network metrics used are given in the Supplementary Material (Supplementary Table S1). More details can been found in a previous report (Rubinov and Sporns, 2010). The calculation of those metrics were implemented in MATLAB (version R2014a, Mathworks Inc., Natick, MA, United States) software with Brain Connectivity Toolbox (Rubinov and Sporns, 2010).

### Statistical Analysis

Statistical evaluations were conducted using R program (version 4.0.2). For cross-sectional comparisons of demographic and clinical characteristics between the MCI and NC groups, the normality of the data was first evaluated by Lilliefors test. The Fisher’s exact test was applied to categorical variables (only gender here), and Wilcoxon rank sum test or *t*-test was used to compare the continuous variables in the case of violating the normality or not. Logistic regression analysis, which considered group as dependent variable and network metrics (both global and nodal measures, i.e., *C*, *L*, *GE*, *SW*, and *nodal bc*_*i*_) as independent variables, was used to evaluate whether there is significant difference of network metrics between both groups. Moreover, gender was controlled as concomitant variable in the logistic regression analysis. *p* < 0.05 was considered as an indicator for significant difference.

### Classification of MCI and NC

In this study, in addition to four scores of cognitive assessments, there are four global and 116 nodal (i.e., *bc*) network metrics for each subject, resulting in 124 features under each sparsity. Considering the classification scenario with high-dimensional features and low-size samples, we hereby proposed a two-layer random forest approach for the task, with the first layer for feature selection and the second for classification. Such an approach was implemented on Python 3.7 with the widely used scikit-learn library (Pedregosa et al., 2011).

The importance of the used features can be measured by the out-of-bag (OOB) error (Genuer et al., 2010). In the random forest approach, under each sparsity, all the 124 features were fed into the first-layer forest and ranked according to the OOB error provided by scikit-learn. Afterward, the top *N* important features of the first layer were selected and fed into the second layer to train a model. A wide range of *N* from 5 to 30 was considered in this study.

For the second layer, two hyperparameters of the random forest, i.e., the number of trees in the forest and the maximum depth of the tree, were fine-tuned. Specifically, a five-fold grid search was embedded in an outer loop to fulfill a 10-fold nested cross-validation (CV) for evaluating the performance of the classifier, i.e., the accuracy, sensitivity, specificity, and area under receiver operating characteristic curve (AUC). Nested CV was demonstrated to produce robust and unbiased performance estimates regardless of sample size (Vabalas et al., 2019).

## Results

### Global Network Metrics of Scale I

**FIGURE 1:**
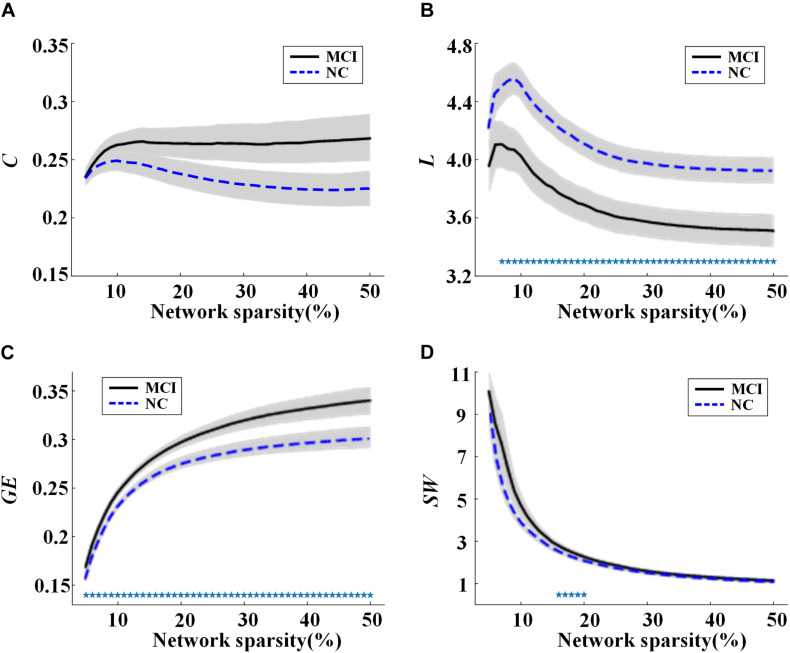
Values of global network metrics (the central line represents the group mean and the envelopes represents mean ± standard error) for normal controls (NC) and mild cognitive impairment (MCI) patients: **(A)** clustering coefficient, **(B)** characteristic path length, **(C)** global efficiency, and **(D)** small worldness. The symbol “*” represents a significant group difference of the network metric in the corresponding sparsity (*p* < 0.05, logistic regression analysis, controlling gender).

Global network properties including *C*, *L*, *GE*, and *SW* were calculated and compared for the MCI and NC groups across sparsities ranging from 5 to 50%, with steps of 1%. The results are illustrated in Figure 1. In both groups, all the global network metrics altered rapidly along with small sparsities and gradually converged toward a sparsity of 50%. No significant group difference of *C* was found at any sparsities (Figure 1A). Compared to NC, a significant decrease (or increase) of *L* (or *GE*) was observed in MCI across almost all the sparsities (Figures 1B,C). As shown in Figure 1D, the SW values were larger than one for both groups under all the calculated sparsities, suggesting the existence of small-world properties in the functional networks. However, significant group difference of *SW* can only be observed with the sparsity of 16–20%.

**FIGURE 2:**
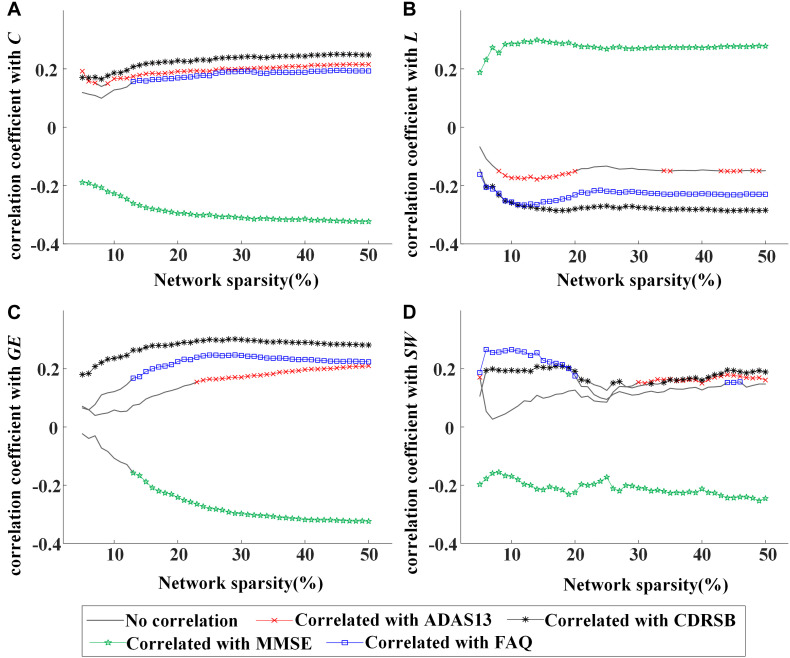
Values of correlation coefficient between global network metrics and clinical assessments [13-item Alzheimer’s disease assessment scale (ADAS13), clinical dementia rating scale sum of boxes (CDRSB), Mini-Mental State Examination (MMSE), and frequently asked questions (FAQ) scores]: **(A)** clustering coefficient, **(B)** characteristic path length, **(C)** global efficiency, and **(D)** small worldness. The symbol “×,” “*,” “*,”or “⋄” represents a significant correlation (*p* < 0.05, partial correlation analysis, controlling gender) between the network metric and cognitive score in the corresponding sparsity.

Moreover, partial correlation analysis (controlling gender) was used to evaluate the association between global network metrics (*C*, *L*, *GE*, and *SW*) and clinical characteristics (ADAS13, CDRSB, MMSE, and FAQ scores) by pooling all participants together. The values of C were shown to be associated negatively with MMSE and positively with CDRSB, ADAS13, or FAQ scores (Figure 2A), indicating a stable association between *C* and clinical symptoms of MCI regardless of network sparsity. Similarly, significant correlation was found between other global network metrics and the cognitive scores.

In Figures 1, 2, rapid alterations can be observed on both the values of the global network metrics and the correlation coefficients between them and the clinical scores when the used network sparsity was small (about <13%). Such an observation may be attributed to the isolated ROIs in the individual networks, which has a percentage >5% when sparsity <9% for NC and 12% for MCI group (shown in Supplementary Figure S1).

### Nodal Characteristics of Scale II

**FIGURE 3:**
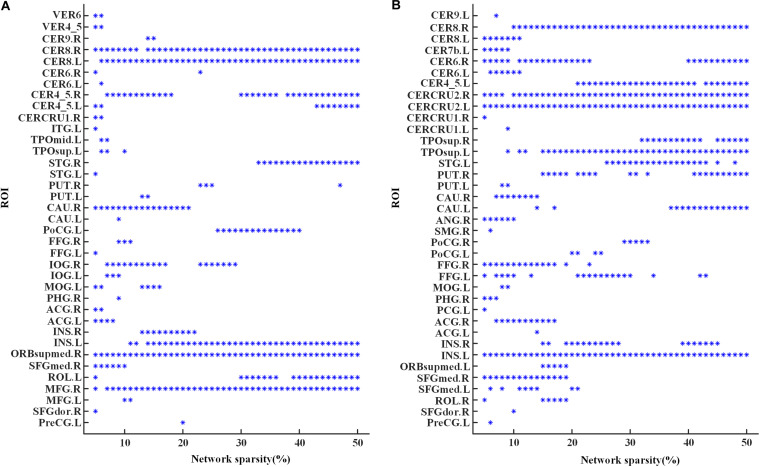
Global hubs of the functional brain networks in **(A)** the MCI group and **(B)** the NC group across the sparsities from 5 to 50%. An “^∗^” symbol indicates that the corresponding region of interest (ROI) (the value of *y*-axis) is identified as a global hub at the corresponding sparistiy (the value of *x*-axis). For abbreviation of ROIs, see Supplementary Table S2.

The global hubs of the functional brain network were detected in both groups across all the considered sparsities. As shown in Figure 3, the right lobule VIII of cerebellar hemisphere (CER8.R) and the left insula (INS.L) were identified as global hubs across almost all the sparsities in both groups. Additionally, global hubs also occur frequently on regions including the left lobule VIII of the cerebellar hemisphere (CER8.L), the right lobule IV–V of the cerebellar hemisphere (CER4-5.R), the right superior frontal gyrus of the medial orbital surface (ORBsupmed.R), and the right middle frontal gyrus (MFG.R) in MCI (Figure 3A), as well as the right lobule VI of the cerebellar hemisphere (CER6.R), the left lobule IV–V of the cerebellar hemisphere (CER4-5.L), the bilateral Crus II of the cerebellar hemisphere (CERCRU2), and the left temporal pole of the superior temporal gyrus (TPOsup.L) in NC (Figure 3B). Moreover, regions in MCI, including the left rolandic operculum (ROL.L), the right superior temporal gyrus (STG.R), the right inferior occipital gyrus (IOG.R), and right caudate nucleus (CAU.R), and regions in NC, such as the right putamen (PUT.R), the left superior temporal gyrus (STG.L), the right temporal pole of superior temporal gyrus (TPOsup.R), and bilateral fusiform gyrus (FFG) were also identified as global hubs at about one third (or more) sparsities.

**FIGURE 4:**
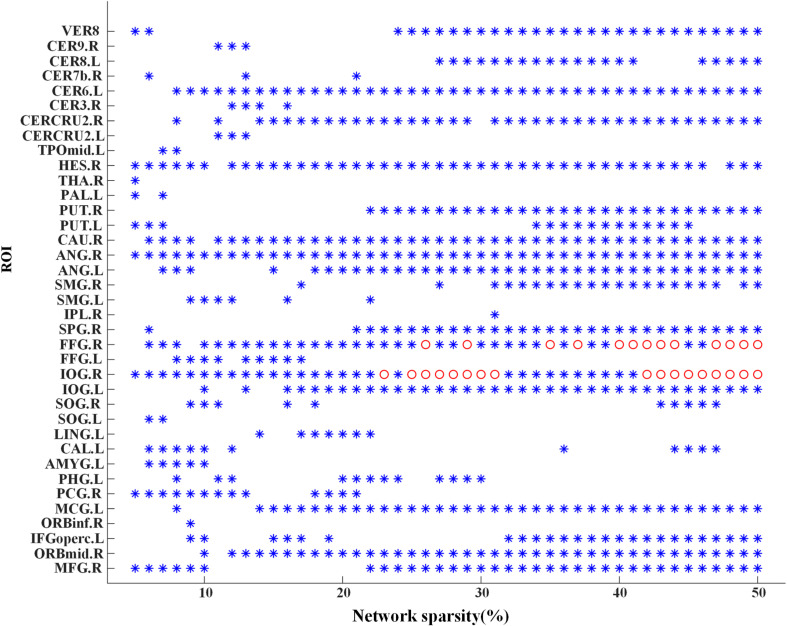
Group difference of nodal betweenness centrality of the individual brain networks across the sparsities from 5 to 50%. A symbol of “^∗^” indicates a significant difference (*p* < 0.05, logistic regression analysis, controlling gender) of bc in the corresponding region of interest (ROI) (the value of *y*-axis) at the corresponding sparsity (the value of *x*-axis). Additionally, the “o” symbol is employed for a significant difference at the level of *p* < 0.05/116 (logistic regression analysis, controlling gender). For abbreviation of ROIs, see Supplementary Table S2.

**FIGURE 5:**
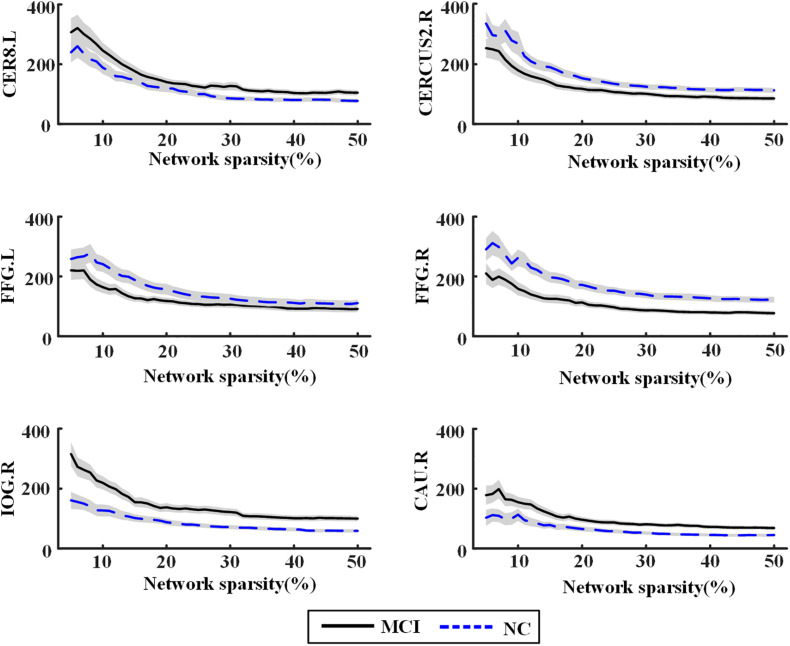
Nodal betweenness centrality of the individual brain networks for normal control (NC) and mild cognitive impairment (MCI) in six regions of interest (ROIs). The central line represents the group mean and the envelopes represents mean ± standard error. For abbreviation of ROIs, see Supplementary Table S2.

Significant differences of nodal *bc* between MCI patients and NCs were found in many ROIs, which were also identified as global hubs and altered across the groups, such as CER8.L, CERCRU2.R, bilateral FFG, IOG.R, and CAU.R (Figure 4). Figure 5 further illustrates the values of *bc* in those regions, demonstrating an agreement with the quantitative information provided by the hubs of the group-level network.

### The Modular Structure of Scale III

**FIGURE 6:**
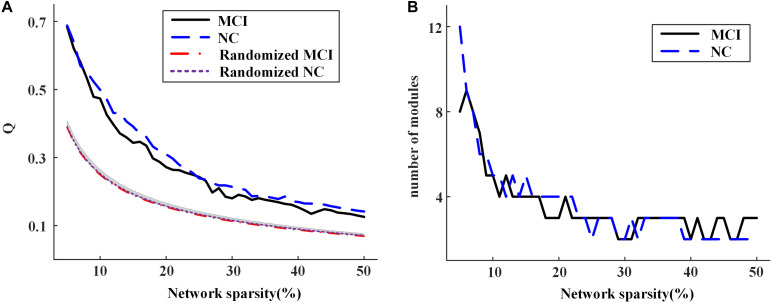
**(A)** The modularity of group-level networks and randomly shuffled networks; **(B)** the number of modules of the community structure detected in the group-level networks for normal control (NC) and mild cognitive impairment (MCI) at different network sparsities.

Figure 6A shows the modularity *Q* achieved for group-level networks and the corresponding randomized networks across the sparsities from 5 to 50%. The value of *Q* decreased with the increasing network sparsity for both groups. The result of permutation tests demonstrated that *Q* is significantly larger than those obtained by the randomly shuffled networks, indicating that the modular structure obtained is non-random across all the sparsities (Figure 6A) (see Supplementary Material for the details of permutation tests). The number of modules detected for each group also alters with the sparsities (Figure 6B). When there are no isolated nodes in the group-level network (i.e., sparsity larger than 7%), there would be two to five modules in each group.

**FIGURE 7:**
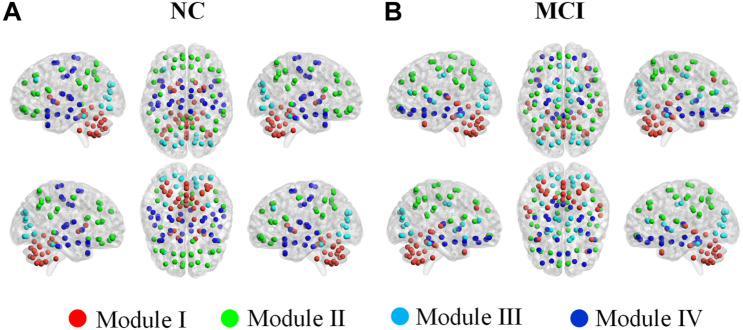
Color online modular structure of functional brain networks for: **(A)** normal controls (NC), **(B)** mild cognitive impairment (MCI) patients. Each dot in the surface representation (BrainNet Viewer, http://www.nitrc. org/projects/bnv/, version 1.61) corresponds to a region of interest. The modular structures were detected based on the group-level network at a sparsity of 16%.

Table 2 and Figure 7 illustrated the modular structures detected in both groups at a sparsity of 16% where we obtained highest performance to discriminate MCI patients from NCs (introduced below). Four functionally oriented modules were uncovered for both groups. For the modular structure of NC, anatomically adjacent ROIs tended to be clustered into the same module. The first module, represented as Module I, contains the thalamus, basal ganglia, and all the ROIs in the cerebellar regions. Module II of NC covers the entire parietal lobe and the majority of frontal lobe. Moreover, the right anterior cingulate and paracingulate gyri, the bilateral precentral gyrus, and the bilateral posterior cingulate gyrus also joined in this module. The left middle frontal gyrus and all the ROIs located in the occipital lobe constitute a new module, named as Module III. All the rest of the ROIs were included in Module IV for NC group. For the MCI group, although anatomically adjacent regions are still likely to be included in the same module, alterations occur in its composition of modules, with a less localized organization of community structure compared with NC. For example, the ROIs of the cerebellum are separated into two modules, whereas they stay in the same module in the NC group. Moreover, the orbital surface of the frontal lobe distributed in two modules and exhibited a denser connection with ROIs such as the bilateral insula, hippocampus, and amygdala.

**TABLE 2:** Alterations of modular composition in MCI group relative to NC.

**Module in NC**	**Anatomical classification (abbreviation) of ROIs**	**Module in MCI**
I (34)	CERCRU1.L, CERCRU1.R, CERCRU2.L, CERCRU2.R, CER4_5.R, CER6.L, CER6.R, CER7b.L, CER7b.R, CER8.L, CER8.R, CER9.L, CER9.R, CER10.L, CER10.R, VER4_5, VER6, VER7, VER8, VER9, VER10	I (21)
	CER3.L, CER3.R, CER4_5.L, VER1_2, VER3	IV (5)
	CAU.L, CAU.R, PUT.L, PUT.R, PAL.L, PAL.R, THA.L, THA.R	III (8)
II (37)	PreCG.L, PreCG.R, SFGdor.L, SFGdor.R, IFGoperc.L, IFGoperc.R, IFGtriang.L, IFGtriang.R, SFGmed.L, SFGmed.R, ORBsupmed.L, ORBsupmed.R, PCG.L, PCG.R, SPG.L, SPG.R, IPL.L, IPL.R, SMG.L, SMG.R, ANG.L, ANG.R, PCUN.L, PCUN.R	II (24)
	MFG.R, MTG.R	I (2)
	ORBsup.L, ORBsup.R, ORBmid.L, ORBmid.R, ORBinf.L, ORBinf.R, REC.L, REC.R	IV (8)
	OLF.R, ACG.L, ACG.R	III (3)
III (15)	CAL.L, CAL.R, CUN.L, CUN.R, LING.L, LING.R, SOG.L, SOG.R, MOG.L, MOG.R, IOG.L, IOG.R, FFG.L, FFG.R	III (14)
	MFG.L	II (1)
IV (30)	STG.L, STG.R, MTG.L, TPOmid.L, TPOmid.R, ITG.L, ITG.R	I (7)
	ROL.L, ROL.R, SMA.L, SMA.R, PoCG.L, PoCG.R, PCL.L, PCL.R	II (8)
	OLF.L, INS.L, INS.R, HIP.L, HIP.R, PHG.L, PHG.R, AMYG.L, AMYG.R, HES.L, HES.R, TPOsup.L, TPOsup.R	IV (13)
	MCG.L, MCG.R	III (2)

*NC, normal controls; MCI, mild cognitive impairment subjects. For the description of the AAL-atlas abbreviations, see Supplementary Table S2.*

### Classification of MCI and NC

**FIGURE 8:**
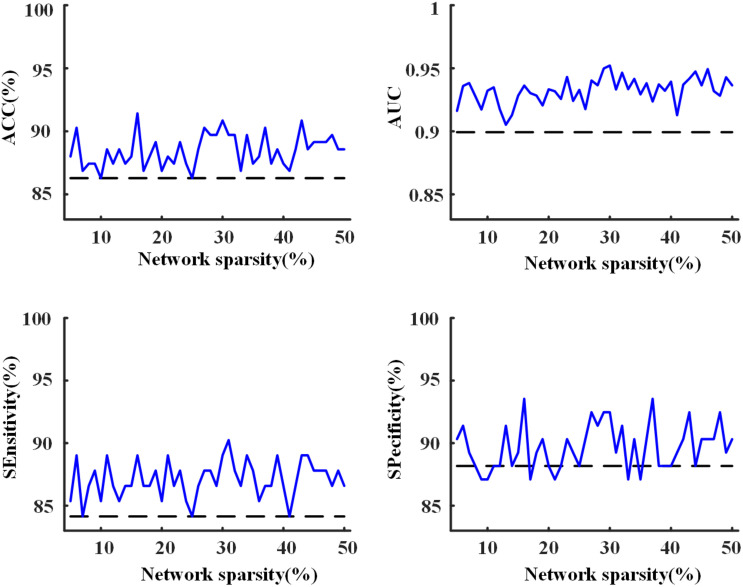
The accuracy, area under receiver operating characteristic curve (AUC), sensitivity, and specificity of the 10-fold cross-validation (CV) for the classification of mild cognitive impairment (MCI) and normal control (NC) with the proposed two-layer random forest approach. The dashed line was achieved by using only the scores of clinical assessments while the solid line represents the model performance obtained after the combination of clinical assessments and network analysis.

Using the proposed two-layer random forest approach, we performed the classification of MCI patients and NCs. An accuracy of 86.3% was obtained by merely using the clinical assessments (scores of MMSE, CDRSB, ADAS13, and FAQ). However, when integrated with network metrics, improved accuracies can be achieved across all the sparsities (as shown in Figure 8), with highest accuracy of 91.4% obtained at the sparsity of 16%. The results suggested that the network metrics could provide additional useful information to assist the diagnosis of MCI patients.

**FIGURE 9:**
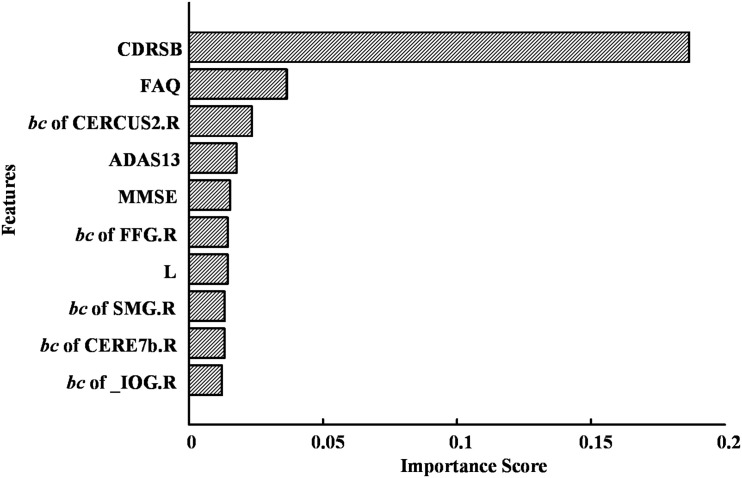
The top 10 discriminative features and their feature importance in the first-layer random forest for the classification of mild cognitive impairment (MCI) and normal control (NC) at a sparsity of 16%. For abbreviation of regions of interest (ROIs), see Supplementary Table S2.

The highest accuracy (91.4%) was achieved when we used the top 10 discriminative features (as shown in Figure 9). The CDRSB was found to be the most informative feature for the classification of MCI patients and NCs, with an overwhelming importance compared with the other features. Other cognitive assessments, FAQ, ADAS13, and MMSE, ranked at the second, fourth, and fifth positions, respectively. As for the network properties, nodal *bc* of CERCRU2.R also ranked in the top 5 important features. Moreover, the top 10 discriminative features included global metric *L* and nodal *bc* of another four regions, i.e., FFG.R, the right supramarginal gyrus (SMG.R), the right lobule VIIB of the cerebellar hemisphere (CERE7b.R), and IOG.R.

## Discussion

In the present work, we investigated the alteration of brain functional network in MCI patients. The network measures were explored on three scales, concerning its global metrics, nodal characteristics, and modular properties. Furthermore, the application of network metrics for patient’s identification was performed and evaluated on a two-layer random forest approach. The results showed significant alterations of network metrics in MCI and suggested that the analysis of brain functional network could provide assistant information for the diagnosis of MCI with neuropsychological assessments.

### Alterations in Global Network Metrics

The global network properties of scale I have been widely investigated in previous studies (Stam and Reijneveld, 2007; Jalili, 2016). However, discrepancies exist in previous studies regarding the alteration of *C*, *L*, and *GE* of the human brain network in MCI. Taking *L* for example, some researchers found no significant difference between MCI and NC (Yao et al., 2010), while others reported a significant increase (Yao et al., 2018) or a significant decrease (Son et al., 2015) of *L* in MCI compared with NC. Here, significantly decreased *L* and increased *GE* were found in MCI compared with NC under almost all the considered sparsities, suggesting that an enhanced functional integration of brain network might occur at the prodromal stage of AD. Such an observation might be indicative of a possible compensatory mechanism in the early stage of AD (Zhou and Lui, 2013). Moreover, existing studies have demonstrated that the clinical symptoms of AD, such as impairments of memory, language, and other cognitive functions, were associated with abnormal structural and functional brain networks (Liu et al., 2017). In this study, we also observed a significant correlation between global network metrics and the clinical cognitive evaluations, suggesting that graph theory analysis could act as a strategy to differentiate MCI patients from NC subjects.

### Alterations in Nodal Characteristics of Scale II

The existence of global hubs in human brain networks was supported by the present study. Although in this study, the global hubs were identified on the group-level network, it provided informative findings that might be associated with the underlying pathological mechanism of MCI.

The most informative observation for the global hubs is its distribution in the cerebellum. For both groups, stable hubs were mainly distributed in cerebellar lobules IV–VI, VII, and VIII. Studies on cerebellar functional topography have shown that activity in sensorimotor regions were related to the contralateral cerebellar lobules IV–VI and VIII, whereas more cognitively demanding tasks engaged prefrontal and parietal cortices along with cerebellar lobules VI and VII (Stoodley et al., 2012). In the present study, CER8.R and CER4_5.L or CER4_5.R were identified as global hubs in both groups, which might suggest a maintained motor function in the MCI group. However, prominent alteration has been revealed within cerebellar lobules VI and VII since bilateral CERCRU2 (a part of lobule VII) and CER6.L were found to be stable hubs in the NC group but not in the MCI group (except for a few sparsities). Furthermore, the nodal *bc* of CERCRU2.R decreased significantly (*p* < 0.05, uncorrected) in MCI patients. We further observed positive correlation (partial correlation analysis, *p* < 0.05, controlling for gender) between nodal *bc* of CRECRU2.L and MMSE score across 40 out of 46 sparsities (average correlation coefficient over these sparsities was 0.19), and negative correlation between nodal *bc* of CERCRU2.R and ADAS13 scores (32 sparsities, average correlation coefficient was −0.18). The MMSE is the best known and the most common used short screening tool of AD for providing an overall measure of cognitive impairment in clinical, research, and community settings (Arevalo-Rodriguez et al., 2015), where ADAS13 is another widely used cognitive assessment with a higher value indicating poorer cognitive performance (Mohs et al., 1997; Sano et al., 2011). In this study, cognitive impairment of the MCI patients was reflected by both MMSE and ADAS13 scores (Table 1). A previous study demonstrated that cognitive impairments may occur when posterior lobe lesions affect cerebellar lobules VI and VII, which would disrupt cerebellar modulation of cognitive loops with cerebral association cortices (Stoodley et al., 2012). Therefore, our findings suggested that alteration of FC in the cerebellum (especially in the CERCRU2) be associated with the cognitive impairment in MCI, and the cerebellum may be a potential target for neuromodulation in treating MCI.

Significant changes of the network metrics of scale II were also in found the bilateral FFG (especially FFG.R) and IOG.R. FFG is thought to be a key structure for functionally specialized computations of high-level vision such as face perception, object recognition, and reading (Weiner and Zilles, 2016). Katja Weibert and Timothy J. Andrews demonstrated that the activity in FFG.R predicts the behavioral advantage for the perception of familiar faces (Weibert and Andrews, 2015). Based on a study of rs-fMRI, Cai et al. (2015) reported altered FC of FFG in patients suffering from amnestic MCI. IOG is also important for the visual function during face processing, since it is connected to the amygdala via white matter connectivity and forms a network for facial recognition with the amygdala (Sato et al., 2017). Previous studies demonstrated activation in bilateral FFG and IOG.R revealed by a face localizer contrast (faces–objects) (Rossion et al., 2003). In the present study, we found that both FFG.R and FFG.L are frequently present as global hubs in NC but absent in MCI, while IOG.R turns out in MCI but not in NC. Furthermore, the nodal *bc* of FFG.R decreased significantly in MCI while that of IOG.R significantly increased. We thus speculated that MCI patients might have an affected function of FFG.R, leading to a compensatory role in IOG.R.

### Alterations in Modular Structure of Scale III

The present study confirms the existence of modular organization in human brain networks, even in MCI patients. We detected four modules for each group. For both groups, although discrepancies existed in their composition of modules, some common features can be found in the modular structure. Such features might throw light on the basis of two fundamental aspects of the human functional brain network, i.e., the functional segmentation and integration. On the one hand, anatomically adjacent ROIs tend to be clustered into the same module, which might be the foundation of the functional segmentation of the brain network. On the other hand, those ROIs, whose anatomical neighbors were involved into a different module, are likely to act as bridges to connect different modules and to be identified as global hubs in the whole network. Such a phenomenon should contribute to the functional integration and the existence of small-worldness property of the brain network.

The prominent alteration of the modular structure in MCI (compared with NC) occurs in the cerebellum, with its ROIs grouped into two modules. Another obvious change in the modular structure in MCI occurs in the medial prefrontal cortex, especially the orbitofrontal cortex (OFC). In previous studies, the OFC has been found to be involved in sensory integration, in representing the affective value of reinforcers, and in decision making and expectation (Kringelbach, 2005). In the present study, six out of eight OFC ROIs are shifted to Module IV and clustered with the hippocampus and parahippocampal regions in MCI. As structural abnormalities in the OFC have been revealed by neuroimaging studies in MCI patients (Wang et al., 2020), in the future, it would be of interest to investigate whether our findings related to the OFC is the cause or effect of its structural abnormalities in MCI.

### The Classification of MCI and NC

In clinical practice, the MCI diagnosis mainly depended on concerns of the cognition changes from the patient, knowledgeable informant, or according to a skilled clinician’s observation (Langa and Levine, 2014). Neuropsychological assessments, such as CDRSB and MMSE, are often used in clinical trial for objective evidence of cognitive impairment (Langa and Levine, 2014). In the present study, with the combination of MMSE, CDRSB, ADAS13, and FAQ scores, we found an accuracy of 86.3% for the classification of MCI and NC, confirming the effectiveness of neuropsychological assessments in the diagnosis of MCI. Improved performances with highest accuracy of 91.4% can be achieved by combining neuropsychological assessments and network analysis after feature selection implemented via random forest approach. Furthermore, we found that the CDRSB score played a vital role in discriminating MCI, in line with a previous study which demonstrated that the CDRSB score could be used to accurately stage severity of AD and MCI (O’bryant et al., 2008). In addition, our results of feature selection further indicated the importance to investigate the role of CERCRU2.R, FFG.R, and IOG.R in MCI.

## Conclusion and Limitation

In this study, we investigated the alterations of brain functional network in MCI. Although small-world properties, global hubs, and modular structures were observed in both groups, network metrics significantly changed in MCI when compared with NC. The role of cerebellar regions, especially the Crus II of cerebellar hemisphere, were found to be associated with the cognitive impairment in MCI patients and discriminative in the identification of MCI. Although network metrics were demonstrated to provide useful information to assist the diagnosis of MCI in clinical practice, future investigation is required to clarify the association between these alterations and the underlying pathological mechanism of MCI.

Moreover, the sex factor was controlled in the statistical analysis to evaluate the group difference of network metrics or their association with clinical characteristics in scales I and II. Because the modular structure of each group was computed at group level, the findings in scale III are hereby descriptive, which suggests that we cannot statistically assess whether they are partly contributed by sex difference. Given our observation that sex contribution to scales I and II is trivial, we speculate that the descriptive modular structure is not contributed by sex difference. Future study on a larger sample is thus in favor of the validation of our findings, especially those in scale III.

## Data Availability Statement

Publicly available datasets were analyzed in this study, which can be found at: http://adni.loni.usc.edu/.

## Ethics Statement

The studies involving human participants were reviewed and approved by the institutional review board of each participating center in the ADNI study. Each participant provided signed informed consent before the study. All methods were carried out in accordance with relevant guidelines and regulations. The current study analyzed de-identified data from the ADNI database, and did not involve a research protocol requiring approval by the relevant institutional review board or ethics committee.

## Author Contributions

FH, JW, JQ, and AY designed this study. LZ and ZY analyzed the data. LZ, FH, and HN wrote the article. All authors contributed to the article and approved the submitted version.

## Conflict of Interest

The authors declare that the research was conducted in the absence of any commercial or financial relationships that could be construed as a potential conflict of interest.

## Footnotes

1 http://adni.loni.usc.edu/

2 http://www.fil.ion.ucl.ac.uk/spm

3 http://rfmri.org/DPABI

## References

[B1] Ahmadlou M. Adeli H. Adeli A. (2010). New diagnostic EEG markers of the Alzheimer’s disease using visibility graph. *J. Neural Transm.* 117 1099–1109. 10.1007/s00702-010-0450-3 20714909

[B2] Al-Shoukry S. Rassem T. H. Makbol N. M. (2020). Alzheimer’s diseases detection by using deep learning algorithms: a mini-review. *IEEE Access* 8 77131–77141. 10.1109/access.2020.2989396

[B3] Arevalo-Rodriguez I. Smailagic N. Roqué I Figuls M. Ciapponi A. Sanchez-Perez E. Giannakou A. (2015). Mini-Mental State Examination (MMSE) for the detection of Alzheimer’s disease and other dementias in people with mild cognitive impairment (MCI). *Cochrane Database Systematic Rev.* 2015:CD010783. 10.1002/14651858.CD010783.pub2 25740785 PMC6464748

[B4] Burgess G. C. Kandala S. Dan N. Laumann T. O. Power J. D. Adeyemo B. (2016). Evaluation of denoising strategies to address motion-correlated artifact in resting state fmri data from the human connectome project. *Brain Connect.* 6 669–680. 10.1089/brain.2016.0435 27571276 PMC5105353

[B5] Cai S. Chong T. Zhang Y. Li J. von Deneen K. M. Ren J. (2015). Altered functional connectivity of fusiform gyrus in subjects with amnestic mild cognitive impairment: a resting-state fMRI study. *Front. Hum. Neurosci.* 9:471. 10.3389/fnhum.2015.00471 26379534 PMC4550786

[B6] Calzà L. Gagliardi G. Rossini Favretti R. Tamburini F. (2021). Linguistic features and automatic classifiers for identifying mild cognitive impairment and dementia. *Comp. Speech Lang.* 65:101113 10.1016/j.csl.2020.101113

[B7] Chen G. Ward B. D. Xie C. (2011). Classification of Alzheimer disease, mild cognitive impairment, and normal cognitive status with large-scale network analysis based on resting-state functional MR imaging. *Radiology* 259 213–221. 10.1148/radiol.10100734 21248238 PMC3064820

[B8] Ciric R. Rosen A. Erus G. Cieslak M. Adebimpe A. Cook P. A. (2018). Mitigating head motion artifact in functional connectivity MRI. *Nat. Protocols* 13 2801–2826. 10.1038/s41596-018-0065-y 30446748 PMC8161527

[B9] Ciric R. Wolf D. H. Power J. D. Roalf D. R. Baum G. L. Ruparel K. (2017). Benchmarking of participant-level confound regression strategies for the control of motion artifact in studies of functional connectivity. *Neuroimage* 154 174–187. 10.1016/j.neuroimage.2017.03.020 28302591 PMC5483393

[B10] Cui X. Xiang J. Guo H. Yin G. Zhang H. Lan F. (2018). Classification of Alzheimer’s disease, mild cognitive impairment, and normal controls with subnetwork selection and graph kernel principal component analysis based on minimum spanning tree brain functional network. *Front. Comput. Neurosci.* 12:31. 10.3389/fncom.2018.00031 29867424 PMC5954113

[B11] Davatzikos C. Bhatt P. Shaw L. M. Batmanghelich K. N. Trojanowski J. Q. (2011). Prediction of MCI to AD conversion, via MRI, CSF biomarkers, and pattern classification. *Neurobiol. Aging* 32:2322.e19-27. 10.1016/j.neurobiolaging.2010.05.023 20594615 PMC2951483

[B12] Deng Y. Shi L. Lei Y. Wang D. Alzheimer’s Disease, and Neuroimaging Initiative. (2016). Altered topological organization of high-level visual networks in Alzheimer’s disease and mild cognitive impairment patients. *Neurosci. Lett.* 630 147–153. 10.1016/j.neulet.2016.07.043 27461791

[B13] Fang J. Chen H. Cao Z. Jiang Y. Ma L. Ma H. (2017). Impaired brain network architecture in newly diagnosed parkinson’s disease based on graph theoretical analysis. *Neurosci. Lett.* 14 151–158. 10.1016/j.neulet.2017.08.002 28789983

[B14] Fornito A. Zalesky A. Breakspear M. (2013). Graph analysis of the human connectome: Promise, progress, and pitfalls. *Neuroimage* 80 426–444. 10.1016/j.neuroimage.2013.04.087 23643999

[B15] Genuer R. Poggi J. M. Tuleau-Malot C. (2010). *Variable Selection Using Random Forests.* Amsterdam: Elsevier Science Inc 10.1016/j.patrec.2010.03.014

[B16] Gosztolya G. Vincze V. Tóth L. Pákáski M. Kálmán J. Hoffmann I. (2019). Identifying mild cognitive impairment and mild Alzheimer’s disease based on spontaneous speech using ASR and linguistic features. *Comp. Speech Lang.* 53 181–197. 10.1016/j.csl.2018.07.007

[B17] He Y. Chen Z. Evans A. (2008). Structural insights into aberrant topological patterns of large-scale cortical networks in Alzheimer’s disease. *J. Neurosci.* 28 4756–4766. 10.1523/jneurosci.0141-08.2008 18448652 PMC6670444

[B18] Jalili M. (2016). Functional brain networks: does the choice of dependency estimator and binarization method matter? *Sci. Rep.* 6:29780. 10.1038/srep29780 27417262 PMC4945914

[B19] Jalili M. (2017). Graph theoretical analysis of Alzheimer’s disease: discrimination of AD patients from healthy subjects. *Inform. Sci.* 384 145–156. 10.1016/j.ins.2016.08.047

[B20] Jie B. Zhang D. Gao W. Wang Q. Wee C. Y. Shen D. (2013). Integration of network topological and connectivity properties for neuroimaging classification. *IEEE Transact. Bio-med. Eng.* 61 576–589. 10.1109/tbme.2013.2284195 24108708 PMC4106141

[B21] Jie B. Zhang D. Wee C.-Y. (2014). Topological graph kernel on multiple thresholded functional connectivity networks for mild cognitive impairment classification. *Hum. Brain Mapp.* 35 2876–2897. 10.1002/hbm.22353 24038749 PMC4116356

[B22] Khazaee A. Ebrahimzadeh A. Babajani-Feremi A. (2015). Identifying patients with Alzheimer’s disease using resting-state fMRI and graph theory. *Clin. Neurophysiol.* 126 2132–2141. 10.1016/j.clinph.2015.02.060 25907414

[B23] Khazaee A. Ebrahimzadeh A. Babajani-Feremi A. (2016). Application of advanced machine learning methods on resting-state fMRI network for identification of mild cognitive impairment and Alzheimer’s disease. *Brain Imag. Behav.* 10 799–817. 10.1007/s11682-015-9448-7 26363784

[B24] Kringelbach M. L. (2005). The human orbitofrontal cortex: linking reward to hedonic experience. *Nat. Rev. Neurosci.* 6 691–702. 10.1038/nrn1747 16136173

[B25] Langa K. M. Levine D. A. (2014). The diagnosis and management of mild cognitive impairment: a clinical review. *JAMA* 312 2551–2561. 10.1001/jama.2014.13806 25514304 PMC4269302

[B26] Li Y. Wang Y. Wu G. Shi F. Zhou L. Lin W. (2011). Discriminant analysis of longitudinal cortical thickness changes in Alzheimer’s disease using dynamic and network features. *Neurobiol. Aging* 33 427.415–430. 10.1016/j.neurobiolaging.2010.11.008 21272960 PMC3086988

[B27] Liu J. Li M. Pan Y. Lan W. Zheng R. Wu F.-X. (2017). Complex brain network analysis and its applications to brain disorders: a survey. *Complexity* 2017:27 10.1017/9781316882610.003

[B28] Meunier D. Achard S. Morcom A. Bullmore E. (2009). Age-related changes in modular organization of human brain functional networks. *Neuroimage* 44 715–723. 10.1016/j.neuroimage.2008.09.062 19027073

[B29] Mohs R. C. Knopman D. Petersen R. C. Ferris S. H. Ernesto C. Grundman M. (1997). Development of cognitive instruments for use in clinical trials of antidementia drugs: additions to the Alzheimer’s disease assessment scale that broaden its scope. the Alzheimer’s disease cooperative study. *Alzheimer Dis. Assoc. Disord* 11 (Suppl. 2), S13–S21. 10.1097/00002093-199700112-00003 9236948

[B30] Murphy K. Bodurka J. Bandettini P. (2007). How long to scan? the relationship between fmri temporal signal to noise ratio and necessary scan duration. *NeuroImage* 34 565–574. 10.1016/j.neuroimage.2006.09.032 17126038 PMC2223273

[B31] Newman M. E. J. (2006). Modularity and community structure in networks. *Proc. Natl. Acad. Sci. U.S.A.* 103 8577–8582. 10.1073/pnas.0601602103 16723398 PMC1482622

[B32] O’bryant S. E. Waring S. C. Cullum C. M. Hall J. Lacritz L. Massman P. J. (2008). Staging dementia using clinical dementia rating scale sum of boxes scores: a texas Alzheimer’s research consortium study. *Arch. Neurol.* 65 1091–1095. 10.1001/archneur.65.8.1091 18695059 PMC3409562

[B33] Onias H. Viol A. Palhano-Fontes F. Andrade K. C. Sturzbecher M. Viswanathan G. (2014). Brain complex network analysis by means of resting state fMRI and graph analysis: will it be helpful in clinical epilepsy? *Epilepsy Behav.* 38 71–80. 10.1016/j.yebeh.2013.11.019 24374054

[B34] Orimaye S. O. Goodkin K. Riaz O. A. Salcedo J. M. Al-Khateeb T. Awujoola A. O. (2020). A machine learning-based linguistic battery for diagnosing mild cognitive impairment due to Alzheimer’s disease. *PLoS One* 15:e0229460. 10.1371/journal.pone.0229460 32134942 PMC7058300

[B35] Pagen L. H. G. Van De Ven V. G. Gronenschild E. H. B. M. Priovoulos N. Verhey F. R. J. Jacobs H. I. L. (2020). Contributions of cerebro-cerebellar default mode connectivity patterns to memory performance in mild cognitive impairment. *J. Alzheimer’s Dis.* 75 633–647. 10.3233/jad-191127 32310164 PMC7458511

[B36] Parkes L. Fulcher B. YüCel M. Fornito A. (2017). An evaluation of the efficacy, reliability, and sensitivity of motion correction strategies for resting-state functional MRI. *Neuroimage* 171 415–436. 10.1016/j.neuroimage.2017.12.073 29278773

[B37] Pedregosa F. Varoquaux G. Gramfort A. Michel V. Thirion B. (2011). Scikit-learn: machine learning in python. *J. Mach. Learn. Res.* 12 2825–2830.

[B38] Pereira J. B. Mijalkov M. Kakaei E. (2016). Disrupted network topology in patients with stable and progressive mild cognitive impairment and Alzheimer’s disease. *Cereb. Cortex* 26 3476–3493. 10.1093/cercor/bhw128 27178195 PMC4961019

[B39] Podhorna J. Krahnke T. Shear M. Harrison J. E. Alzheimer’s Disease, and Neuroimaging Initiative. (2016). Alzheimer’s disease assessment scale–cognitive subscale variants in mild cognitive impairment and mild Alzheimer’s disease: change over time and the effect of enrichment strategies. *Alzheimers Res. Ther.* 8:8. 10.1186/s13195-016-0170-5 26868820 PMC4751673

[B40] Power J. D. Barnes K. A. Snyder A. Z. Schlaggar B. L. Petersen S. E. (2012). Spurious but systematic correlations in functional connectivity MRI networks arise from subject motion. *NeuroImage* 59 2142–2154. 10.1016/j.neuroimage.2011.10.018 22019881 PMC3254728

[B41] Radicchi F. Castellano C. Cecconi F. Loreto V. Parisi D. (2004). Defining and identifying communities in networks. *Proc. Natl. Acad. Sci. U.S.A.* 101 2658–2663. 10.1073/pnas.0400054101 14981240 PMC365677

[B42] Rossion B. Schiltz C. Crommelinck M. (2003). The functionally defined right occipital and fusiform “face areas” discriminate novel from visually familiar faces. *Neuroimage* 19 877–883. 10.1016/S1053-8119(03)00105-8 12880816

[B43] Rubinov M. Sporns O. (2010). Complex network measures of brain connectivity: uses and interpretations. *NeuroImage* 52 1059–1069. 10.1016/j.neuroimage.2009.10.003 19819337

[B44] Sánchez-Catasús C. A. Willemsen A. Boellaard R. Juarez-Orozco L. E. Samper-Noa J. Aguila-Ruiz A. (2018). Episodic memory in mild cognitive impairment inversely correlates with the global modularity of the cerebral blood flow network. *Psychiatry Res. Neuroimag.* 282 73–81. 10.1016/j.pscychresns.2018.11.003 30419408

[B45] Sano M. Raman R. Emond J. Juarez-Orozco L. E. Samper-Noa J. Aguila-Ruiz A. (2011). Adding delayed recall to the Alzheimer disease assessment scale is useful in studies of mild cognitive impairment but not Alzheimer disease. *Alzheimer Dis. Assoc. Disord* 25 122–127. 10.1097/wad.0b013e3181f883b7 20921876 PMC3526369

[B46] Sato W. Kochiyama T. Uono S. Matsuda K. Usui K. Usui N. (2017). Bidirectional electric communication between the inferior occipital gyrus and the amygdala during face processing. *Hum. Brain Mapp.* 38 4511–4524. 10.1002/hbm.23678 28573679 PMC6867177

[B47] Seo E. H. Lee D. Y. Lee J.-M. Park J. S. Sohn B. K. Lee D. S. (2013). Whole-brain functional networks in cognitively normal, mild cognitive impairment, and Alzheimer’s disease. *PLoS One* 8:e53922. 10.1371/journal.pone.0053922 23335980 PMC3545923

[B48] Son S.-J. Kim J. Seo J. Lee J. M. Park H. Alzheimer’s Disease (2015). Connectivity analysis of normal and mild cognitive impairment patients based on FDG and PiB-PET images. *Neurosci. Res.* 98 50–58. 10.1016/j.neures.2015.04.002 25896866

[B49] Stam C. J. Reijneveld J. C. (2007). Graph theoretical analysis of complex networks in the brain. *Nonlinear Biomed. Phys.* 1:3. 10.1186/1753-4631-1-3 17908336 PMC1976403

[B50] Stoodley C. J. (2012). The cerebellum and cognition: evidence from functional imaging studies. *Cerebellum* 11 352–365. 10.1007/s12311-011-0260-7 21373864

[B51] Stoodley C. J. Valera E. M. Schmahmann J. D. (2012). Functional topography of the cerebellum for motor and cognitive tasks: an fMRI study. *NeuroImage* 59 1560–1570. 10.1016/j.neuroimage.2011.08.065 21907811 PMC3230671

[B52] Suk H.-I. Wee C.-Y. Shen D. (2013). Discriminative group sparse representation for mild cognitive impairment classification. *Mach. Learn. Med. Imag.* 2013 131–138. 10.1007/978-3-319-02267-3_17

[B53] Sun Y. Yin Q. Fang R. Yan X. Wang Y. Bezerianos A. (2014). Disrupted functional brain connectivity and its association to structural connectivity in amnestic mild cognitive impairment and Alzheimer’s disease. *PLoS One* 9:e96505. 10.1371/journal.pone.0096505 24806295 PMC4013022

[B54] Tabatabaei-Jafari H. Walsh E. Shaw M. E. Cherbuin N. Alzheimer’s Disease, and Neuroimaging Initiative. (2017). The cerebellum shrinks faster than normal ageing in Alzheimer’s disease but not in mild cognitive impairment. *Hum. Brain Mapp.* 38 3141–3150. 10.1002/hbm.23580 28321950 PMC5426955

[B55] Tanveer M. Richhariya B. Khan R. U. Rashid A. H. Khanna P. Prasad M. (2020). Machine learning techniques for the diagnosis of Alzheimer’s disease: a review. *ACM Trans. Multimedia Comput. Commun. Appl.* 16 1–35. 10.1145/3344998

[B56] Tijms B. M. Wink A. M. De Haan W. van der Flier W. M. Stam C. J. Scheltens P. (2013). Alzheimer’s disease: connecting findings from graph theoretical studies of brain networks. *Neurobiol. Aging* 34 2023–2036. 10.1016/j.neurobiolaging.2013.02.020 23541878

[B57] Vabalas A. Gowen E. Poliakoff E. Casson A. J. (2019). Machine learning algorithm validation with a limited sample size. *PLoS One* 14:e0224365. 10.1371/journal.pone.0224365 31697686 PMC6837442

[B58] Wang J. Zuo X. Dai Z. (2013). Disrupted functional brain connectome in individuals at risk for Alzheimer’s disease. *Biol. Psychiatry* 73 472–481. 10.1016/j.biopsych.2012.03.026 22537793

[B59] Wang Z. Zhang X. Liu R. Wang Y. Qing Z. Lu J. (2020). Altered sulcogyral patterns of orbitofrontal cortex in patients with mild cognitive impairment. *Psychiatry Res. Neuroimag.* 302:111108. 10.1016/j.pscychresns.2020.111108 32464534

[B60] Wee C.-Y. Li Y. Jie B. Peng Z. W. Shen D. (2013a). Identification of MCI using optimal sparse MAR modeled effective connectivity networks. *Med. Image Comput. Comp. Ass. Intervent. – MICCAI* 2013 319–327. 10.1007/978-3-642-40763-5_40 PMC4089866 24579156

[B61] Wee C.-Y. Yap P.-T. Zhang D. Wang L. Shen D. (2013b). Group-constrained sparse fMRI connectivity modeling for mild cognitive impairment identification. *Brain Struct. Funct.* 219 641–656. 10.1007/s00429-013-0524-8 23468090 PMC3710527

[B62] Weibert K. Andrews T. J. (2015). Activity in the right fusiform face area predicts the behavioural advantage for the perception of familiar faces. *Neuropsychologia* 75 588–596. 10.1016/j.neuropsychologia.2015.07.015 26187507

[B63] Weiner K. S. Zilles K. (2016). The anatomical and functional specialization of the fusiform gyrus. *Neuropsychologia* 83 48–62. 10.1016/j.neuropsychologia.2015.06.033 26119921 PMC4714959

[B64] Xiang J. Guo H. Cao R. Liang H. Chen J. (2013). An abnormal resting-state functional brain network indicates progression towards Alzheimer’s disease. *Neural Regenerat. Res.* 8:2789. 10.3969/j.issn.1673-5374.2013.30.001 PMC4146017 25206600

[B65] Xu X. Li W. Mei J. Tao M. Wang X. Zhao Q. (2020). Feature selection and combination of information in the functional brain connectome for discrimination of mild cognitive impairment and analyses of altered brain patterns. *Front. Aging Neurosci.* 12:28. 10.3389/fnagi.2020.00028 32140102 PMC7042199

[B66] Yan C. Zang Y. (2010). DPARSF: a MATLAB toolbox for “pipeline” data analysis of resting-state fMRI. *Front. Systems Neurosci.* 4:13. 10.3389/fnsys.2010.00013 20577591 PMC2889691

[B67] Yan C. G. Wang X. D. Zuo X. N. Zang Y. F. (2016). DPABI: data processing & analysis for (Resting-State) brain imaging. *Neuroinformatics* 14 339–351. 10.1007/s12021-016-9299-4 27075850

[B68] Yao Z. Hu B. Chen X. Xie Y. Gutknecht J. Majoe D. (2018). Learning metabolic brain networks in MCI and AD by robustness and leave-one-out analysis: an FDG-PET study. *Am. J. Alzheimer’s Dis. Other Dementias§* 33 42–54. 10.1177/1533317517731535 28931302 PMC10852436

[B69] Yao Z. Zhang Y. Lin L. Zhou Y. Xu C. Jiang T. (2010). Abnormal cortical networks in mild cognitive impairment and Alzheimer’s disease. *PLoS Comput. Biol.* 6:e1001006. 10.1371/journal.pcbi.1001006 21124954 PMC2987916

[B70] Zhao X. Liu Y. Wang X. Liu B. Xi Q. Guo Q. (2012). Disrupted small-world brain networks in moderate Alzheimer’s disease: a resting-state FMRI study. *PLoS One* 7:e33540. 10.1371/journal.pone.0033540 22457774 PMC3311642

[B71] Zhou Y. Lui Y. W. (2013). Small-world properties in mild cognitive impairment and early Alzheimer’s disease: a cortical thickness MRI study. *ISRN Geriatrics* 2013:542080. 10.1155/2013/542080 25414852 PMC4235771

[B72] Zhuo J. Fan L. Liu Y. Zhang Y. Yu C. Jiang T. (2016). Connectivity profiles reveal a transition subarea in the parahippocampal region that integrates the anterior temporal-posterior medial systems. *J. Neurosci.* 36 2782–2795. 10.1523/jneurosci.1975-15.2016 26937015 PMC6604873

