# Supplementary material for: Investigation on the Alteration of Brain Functional Network and Its Role in the Identification of Mild Cognitive Impairment
Source: Front Neurosci. 2020 Sep 30;14:558434. doi: 10.3389/fnins.2020.558434 (PMC7556272; doi:10.3389/fnins.2020.558434)
Supplement: Supplementary file 1 [file Data_Sheet_1.docx]

**Permutation test for the non-random modularity of the group-level networks**

Similar with previous studies (He, Chen, & Evans, 2008; Wu et al., 2012; Yao et al., 2010), a permutation test was applied to the null hypothesis that modularity (denoted as Q) of community structure achieved in a group-level network is higher than that obtained by chance. We randomly reallocated the edges of a group-level network, redetected its community structure, and recomputed the Q value. This randomization procedure was repeated 999 times and get 999 randomized Q values. Along with the original Q value, the 1000 values were sorted in a descending order and if the original Q falls into the top 5%, the null hypothesis was accepted with a probability of type I error of 0.05. The procedure was repeated at each sparsity of the brain networks.


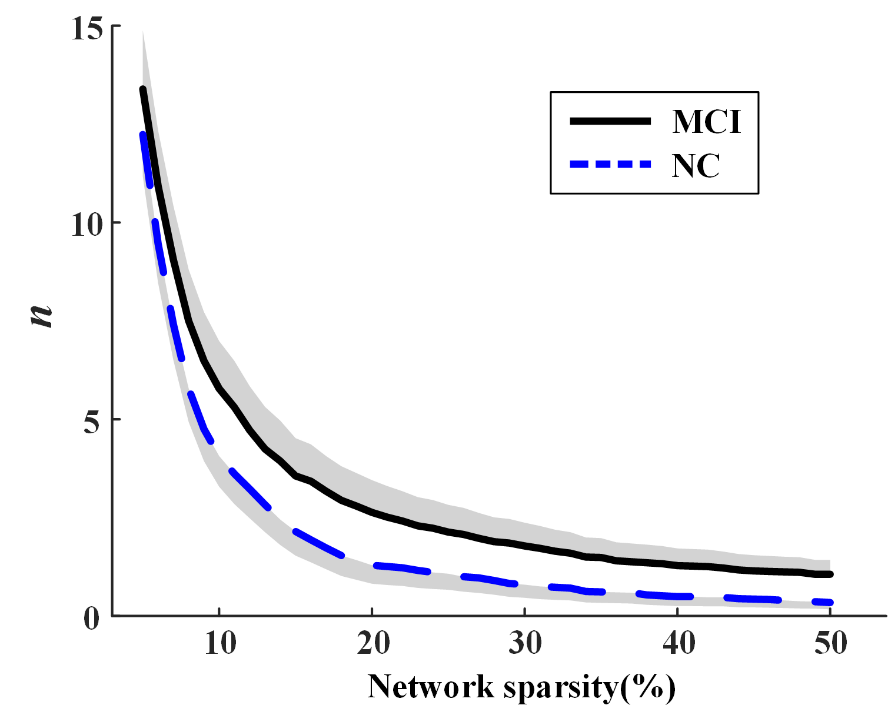


**FIGURE S1 |** Number of isolated ROIs in the individual networks when network sparsity varied from 5% to 50%. The central line represents the group mean, while the envelope represents mean plus standard error for MCI and mean minus standard error for NC, for clarity.

**Table S1|** Definitions and descriptions of network topological metrics used in this study (Rubinov & Sporns, 2010). Here, weighted and undirected network was used.

| Metrics | Definition | Discription |
| --- | --- | --- |
| Characteristic path length (*L*) | $L=\frac{1}{N(N-1)} \sum_{i=1}^{N} \sum_{j\neq i}^{N} {d_{ij}}^{w}$ | Here$, d_{ij}^{w}$is the shortest weighted path length between node *i* and node *j* in a weighted network. |
| Clustering coefficient (*C*) | $C=\frac{1}{N}\sum_{i=1}^{N} \frac{\sum_{j,h=1}^{N} \left( w_{ij}w_{ih}w_{jh} \right)^{\frac{1}{3}}}{k_{i}^{w}(k_{i}^{w}-1)}$ | Here, $w_{ij}$is the weight between node *i* and *j*. $k_{i}^{w} i$s the degree of node *i* (the sum of weight between node *i* and its neighbors). |
| Global efficiency (*GE*) | $GE=\frac{1}{N(N-1)}{\sum_{i=1}^{N} \sum_{j\neq i}^{N} {{(d}_{ij}}^{w})}^{-1}$ | The definition of $d_{ij}^{w}$is the same as aforementioned. |
| Small-worldness (*SW*) | $SW=\frac{C/{C_{rand}}}{L/{L_{rand}}}$ | For the calculation the SW of a network, we need to shuffle the edges in the network randomly for *n* times, leading to *n* random networks (in the present study, *n* was set as 1000). *C_rand_* and *L_rand_* represent for the averaged clustering coefficient and characteristic path length, respectively. Meanwhile, *C* and *L* represent for the clustering coefficient and characteristic path length of the original network. |
| Betweenness centrality (*bc*) | ${bc}_{i}=\frac{1}{(n-1)(n-2)}\sum_{\begin{aligned} h,j\in N \\ h\neq j,h\neq i, \\ j\neq i \end{aligned}} \frac{\rho_{hj}^{(i)}}{\rho_{hj}}$ | The *bc*_i_ is the betweenness centrality of node *i*. Here, $\rho_{hj}$ is the number of shortest paths between node *h* and *j*, and $\rho_{hj}^{(i)}$ is the number of shortest paths between h and j that passes through node *i*. |
| Modularity (*Q*) | $Q=\frac{1}{l^{w}}\sum_{i,j=1}^{N} \left[ w_{ij}-\frac{k_{i}^{w}k_{j}^{w}}{l^{w}} \right]\delta_{m_{i}, m_{j}}$ | $l^{w}$is the total degree of the network (the sum of weight of all the edges in the network). Moreover, as same as with the definition of clustering coefficient, $w_{ij}$is the weight between node *i* and *j* while $k_{i}^{w} i$s the degree of node *i* (the sum of weight between node *i* and its neighbors). $m_{i}$is the module where node *i* belongs to. If *m_i_* equals to *m_j_*, set $\delta_{m_{i}, m_{j}}$as 1 and 0 otherwise. |

**Table S2**| List of the Anatomical Regions of Interest defined in Each Hemisphere (Tzourio-Mazoyer et al., 2002) (Rolls, Huang, Lin, Feng, & Joliot, 2020) and their abbreviations used in the present study.

| Anatomical description | Abbreviations |
| --- | --- |
| Central region | |
| Precentral gyrus | PreCG |
| Postcentral gyrus | PoCG |
| Rolandic operculum | ROL |
| Frontal lobe | |
| Lateral surface | |
| Superior frontal gyrus, dorsolateral | SFGdor |
| Middle frontal gyrus | MFG |
| Inferior frontal gyrus, opercular part | IFGoperc |
| Inferior frontal gyrus, triangular part | IFGtriang |
| Medial surface | |
| Superior frontal gyrus, medial | SFGmed |
| Supplementary motor area | SMA |
| Paracentral lobule | PCL |
| Orbital surface | |
| Superior frontal gyrus, orbital part | ORBsup |
| Superior frontal gyrus, medial orbital | ORBsupmed |
| Middle frontal gyrus, orbital part | ORBmid |
| Inferior frontal gyrus, orbital part | ORBinf |
| Gyrus rectus | REC |
| Olfactory cortex | OLF |
| Temporal lobe | |
| Lateral surface | |
| Superior temporal gyrus | STG |
| Heschl gyrus | HES |
| Middle temporal gyrus | MTG |
| Inferior temporal gyrus | ITG |
| Parietal lobe | |
| Lateral surface | |
| Superior parietal gyrus | SPG |
| Inferior parietal, but supramarginal and angular gyri | IPL |
| Angular gyrus | ANG |
| Supramarginal gyrus | SMG |
| Medial surface | |
| Precuneus | PCUN |
| Occipital lobe | |
| Lateral surface | |
| Superior occipital gyrus | SOG |
| Middle occipital gyrus | MOG |
| Inferior occipital gyrus | IOG |
| Medial and inferior surfaces | |
| Cuneus | CUN |
| Calcarine fissure and surrounding cortex | CAL |
| Lingual gyrus | LING |
| Fusiform gyrus | FFG |
| Limbic lobe | |
| Temporal pole: superior temporal gyrus | TPOsup |
| Temporal pole: middle temporal gyrus | TPOmid |
| Anterior cingulate and paracingulate gyri | ACG |
| Median cingulate and paracingulate gyri | MCG |
| Posterior cingulate gyrus | PCG |
| Hippocampus | HIP |
| Parahippocampal gyrus | PHG |
| Insula | INS |
| Sub cortical gray nuclei | |
| Amygdala | AMYG |
| Caudate nucleus | CAU |
| Lenticular nucleus, putamen | PUT |
| Lenticular nucleus, pallidum | PAL |
| Thalamus | THA |
| Cerebellum | |
| Crus I of cerebellar hemisphere | CERCRU1 |
| Crus II of cerebellar hemisphere | CERCRU2 |
| Lobule III of cerebellar hemisphere | CER3 |
| Lobule IV, V of cerebellar hemisphere | CER4_5 |
| Lobule VI of cerebellar hemisphere | CER6 |
| Lobule VIIB of cerebellar hemisphere | CER7b |
| Lobule VIII of cerebellar hemisphere | CER8 |
| Lobule IX of cerebellar hemisphere | CER9 |
| Lobule X of cerebellar hemisphere | CER10 |
| Lobule I, II of vermis | VER1_2 |
| Lobule III of vermis | VER3 |
| Lobule IV, V of vermis | VER4_5 |
| Lobule VI of vermis | VER6 |
| Lobule VII of vermis | VER7 |
| Lobule VIII of vermis | VER8 |
| Lobule IX of vermis | VER9 |
| Lobule X of vermis | VER10 |

**REFERENCES**

He, Y., Chen, Z., & Evans, A. (2008). Structural insights into aberrant topological patterns of large-scale cortical networks in Alzheimer's disease. *J Neurosci*(No.18), 4756-4766.

Rolls, E. T., Huang, C. C., Lin, C. P., Feng, J., & Joliot, M. (2020). Automated anatomical labelling atlas 3. *Neuroimage, 206*, 116189. doi:10.1016/j.neuroimage.2019.116189

Rubinov, M., & Sporns, O. (2010). Complex network measures of brain connectivity: uses and interpretations. *Neuroimage, 52*(3), 1059-1069. doi:10.1016/j.neuroimage.2009.10.003

Tzourio-Mazoyer, N., Landeau, B., Papathanassiou, D., Crivello, F., Etard, O., Delcroix, N., . . . Joliot, M. (2002). Automated anatomical labeling of activations in SPM using a macroscopic anatomical parcellation of the MNI MRI single-subject brain. *Neuroimage, 15*(1), 273-289. doi:10.1006/nimg.2001.0978

Wu, K., ORCID --- . Department of Nuclear Medicine, Radiology, I. o. D., Aging, Cancer, T. U., Sendai, Japan, kaiwu@idac.tohoku.ac.jp, Taki, Y. D. o. N. M., Radiology, I. o. D., Aging, Cancer, T. U., Sendai, Japan, . . . Cancer, T. U., Sendai, Japan. (2012). Age-related changes in topological organization of structural brain networks in healthy individuals. *Hum Brain Mapp*(No.3), 552-568.

Yao, Z., Zhang, Y., Lin, L., Zhou, Y., Xu, C., Jiang, T., & Initiative, A. s. D. N. (2010). Abnormal cortical networks in mild cognitive impairment and Alzheimer's disease. *PLoS Comput Biol, 6*(11), e1001006.
